# Clinical value of neutrophil-to-lymphocyte ratio and D-dimer in predicting the efficacy of minocycline for pediatric Mycoplasma pneumoniae pneumonia

**DOI:** 10.3389/fped.2026.1865687

**Published:** 2026-09-17

**Authors:** Rui Fang Chen, Xue Jiang, XinYing Zhou, QianYu Zhai, JunLi Wang, Shuo Feng

**Affiliations:** Tianjin Third Central Hospital, Tianjin, China

**Keywords:** biomarkers, D-dimer, neutrophil-to-lymphocyte ratio, pediatric mycoplasma pneumoniae pneumonia, tetracyclines

## Abstract

**Background:**

Mycoplasma pneumoniae pneumonia (MPP) is a common respiratory infection in children. With the increasing prevalence of macrolide-resistant strains, minocycline has become a crucial alternative treatment. However, there is a lack of early predictive markers for evaluating early clinical response. This study aimed to investigate the clinical value of the neutrophil-to-lymphocyte ratio (NLR) and D-dimer levels in predicting early clinical response to minocycline in pediatric MPP.

**Methods:**

A retrospective analysis was conducted on 86 children aged 8–14 years with MPP who received minocycline treatment at Tianjin University Central Hospital between January 2023 and January 2025. Treatment efficacy was defined based on the body temperature response within 48 h after initiating minocycline: patients whose body temperature returned to normal (≤37.3 °C) were classified as having effective treatment (responsive group, *n* = 51), whereas those with persistent fever requiring the addition of systemic corticosteroids (methylprednisolone) were classified as having ineffective treatment (non-responsive group, *n* = 35). Baseline NLR and D-dimer levels, along with other laboratory indicators obtained prior to treatment, were compared between the two groups to evaluate their predictive value for treatment outcome. Multivariate logistic regression and receiver operating characteristic (ROC) curve analyses were performed.

**Results:**

The non-responsive group exhibited significantly higher NLR (median 2.62 vs. 1.69, *P* < 0.001) and D-dimer levels (median 0.36 vs. 0.18 mg/L, *P* < 0.001) compared to the responsive group. Multivariate logistic regression identified NLR (OR=2.113, 95% CI: 1.184–3.770, *P* = 0.011) and D-dimer (OR = 148.867, 95% CI: 4.994–4438.007, *P* = 0.004) as independent risk factors for poor treatment response. ROC analysis demonstrated that the area under the curve (AUC) for the combined detection of NLR and D-dimer was 0.858 (95% CI: 0.756–0.926), which was superior to NLR (AUC=0.773) or D-dimer (AUC=0.776) alone. Optimal cut-off values were determined as 1.904 for NLR (sensitivity 82.9%, specificity 76.7%) and 0.175 mg/L for D-dimer (sensitivity 94.3%, specificity 89.5%). Furthermore, both NLR and D-dimer were positively correlated with the length of hospital stay (r = 0.383 and r = 0.477, respectively, *P* < 0.001).

**Conclusion:**

NLR and D-dimer are reliable and accessible biomarkers for predicting early clinical response to minocycline in pediatric MPP. Elevated baseline levels identify patients at risk for persistent fever and need for treatment escalation. Their combined detection offers high clinical value, facilitating early identification of poor responders and timely adjustment of therapeutic strategies.

## 1 Introduction

Mycoplasma pneumoniae is a major pathogen responsible for community-acquired pneumonia (CAP) in children, accounting for 10% to 40% of pediatric CAP hospitalizations (1). In recent years, particularly following the normalization of COVID-19 pandemic prevention and control measures, widespread outbreaks of Mycoplasma pneumoniae pneumonia (MPP) have been reported globally, posing a significant threat to children's health (2). Macrolides have traditionally been the first-line treatment for pediatric MPP. However, their extensive use has led to a dramatic increase in macrolide-resistant Mycoplasma pneumoniae (MRMP) strains, particularly in Asia, resulting in decreased clinical efficacy and prolonged disease courses (3). Epidemiological surveillance data indicate that the prevalence of MRMP has reached alarmingly high levels, particularly in East Asian countries. In China, macrolide resistance rates among M. pneumoniae isolates from pediatric patients have been reported to exceed 80% in multiple regional studies, with some centers reporting rates as high as 90%–97% (4, 5). Similarly, studies from Japan and South Korea have documented MRMP prevalence rates ranging from 30% to over 90% during epidemic periods (6, 7). The high and increasing prevalence of MRMP directly contributes to treatment failure with first-line macrolide therapy, resulting in prolonged fever, extended hospitalization, and an increased risk of progression to severe or refractory disease. This epidemiological reality underscores the urgent need for alternative therapeutic agents such as minocycline, as well as reliable biomarkers to monitor their treatment efficacy in a timely manner. To address the challenge of MRMP, minocycline has emerged as an effective alternative therapy. This agent exhibits potent antimicrobial activity against Mycoplasma pneumoniae by inhibiting protein synthesis and is currently recommended by international guidelines for children over 8 years of age (8). Due to its relatively short history of clinical application in pediatrics, the development of resistance to minocycline remains slow. Nevertheless, a subset of patients still exhibits poor responses to minocycline monotherapy, progressing to severe or refractory MPP that necessitates adjunctive systemic corticosteroids to control excessive inflammation (9). Currently, there is a critical lack of precise and convenient biomarkers to evaluate and predict early clinical response to minocycline, which often leads to delayed treatment adjustments.

MPP is characterized not only by direct pathogen-induced damage but also by an intense host immune-inflammatory response and coagulation abnormalities. D-dimer, a degradation product of cross-linked fibrin, reflects the activation state of the coagulation and fibrinolytic systems. Elevated D-dimer levels have been strongly associated with the severity of pediatric MPP and the risk of microvascular thrombosis (10). Concurrently, the neutrophil-to-lymphocyte ratio (NLR), an easily calculable parameter from routine blood counts, serves as a potential marker of systemic inflammation. Neutrophilia indicates an active inflammatory response, while lymphopenia reflects immune suppression or stress-induced apoptosis. NLR has shown promise in assessing disease severity and prognosis in various types of pneumonia (11).

Despite their individual prognostic values, the specific roles of NLR and D-dimer in evaluating early clinical response to minocycline for pediatric MPP remain unexplored. This study aims to investigate the clinical utility of NLR and D-dimer, both individually and in combination, in predicting early clinical response to minocycline in children with MPP.

## 2 Materials and methods

### 2.1 Study population

This retrospective study was conducted at Tianjin University Central Hospital, a tertiary pediatric center in Tianjin, China. The study protocol was reviewed and approved by the Institutional Ethics Committee (Approval No. SZX-IIT-2025-41). During the study period (January 2023 to January 2025), a total of 4,198 pediatric patients were admitted to our department. Patient data were retrieved from the hospital's electronic medical record (EMR) system. Two investigators independently reviewed all identified cases to verify eligibility. After systematic review, 86 patients met all eligibility criteria and were included in the final analysis. Given the retrospective design and use of de-identified data, the Ethics Committee waived the requirement for individual informed consent.

MPP was diagnosed based on: (1) clinical manifestations of pneumonia (fever, cough, pulmonary rales); (2) radiographic evidence on chest x-ray or CT; and (3) laboratory confirmation of M. pneumoniae infection, defined as positive M. pneumoniae IgM antibody, or a 4-fold increase in IgG titer in paired sera, or positive M. pneumoniae DNA by PCR from nasopharyngeal swabs (12). Macrolide resistance testing was not routinely performed at our institution during the study period.

Patients were excluded if they had: (1) mild upper respiratory tract infections without radiographic evidence of pneumonia; (2) concurrent respiratory diseases; (3) underlying conditions affecting blood cell counts; or (4) use of immunomodulators or systemic corticosteroids within 14 days prior to admission.

### 2.2 Treatment and grouping

All enrolled patients received minocycline as the primary antimicrobial therapy. The standard dosing regimen was: loading dose of 2 mg/kg (maximum 100 mg) every 12 h for the first 24 h, followed by a maintenance dose of 2 mg/kg (maximum 100 mg) once daily. Prior to minocycline initiation, all patients had received empirical macrolide therapy (azithromycin 10 mg/kg/day for 3–5 days) but demonstrated inadequate clinical response with persistent fever. Patients were classified into two groups: the responsive group (*n* = 51), defined as patients whose body temperature returned to normal (≤37.3 °C) within 48 h; and the non-responsive group (*n* = 35), defined as patients with persistent fever at 48 h despite minocycline therapy, requiring the addition of systemic corticosteroids (methylprednisolone 1–2 mg/kg/day) (13). The terms “responsive” and “non-responsive” are used throughout this manuscript to accurately reflect this classification rationale.

### 2.3 Data collection and statistical analysis

Laboratory parameters were collected from venous blood samples obtained within 24 h of admission, prior to minocycline therapy. The NLR was calculated by dividing the absolute neutrophil count by the absolute lymphocyte count. The coefficient of variation (COV) for NLR was 41.6% in the responsive group and 57.5% in the non-responsive group, while D-dimer showed higher variability with COV values of 60.9% and 115.0%, respectively.

Statistical analyses were performed using SPSS 25.0 software. Continuous variables with non-normal distributions were presented as medians with interquartile ranges (IQR, Q1–Q3), and group comparisons were conducted using the Mann–Whitney U test. Multivariate logistic regression was performed to identify independent predictors. ROC curves were constructed and optimal cut-off values were determined using the Youden index. *P* < 0.05 was considered statistically significant.

## 3 Results

### 3.1 Baseline characteristics

After systematic review, 86 patients were included in the final analysis, comprising 51 in the responsive group and 35 in the non-responsive group. There were no significant differences in age (median 10.0 vs. 10.0 years, *P* = 0.367) and gender distribution (*P* = 0.434). However, the length of hospital stay was significantly longer in the non-responsive group (median 8.0 vs. 5.0 days, *P* < 0.001) (Figure 1). Detailed blood cell analysis parameters are presented in Supplementary Table S3.

**Figure 1:**
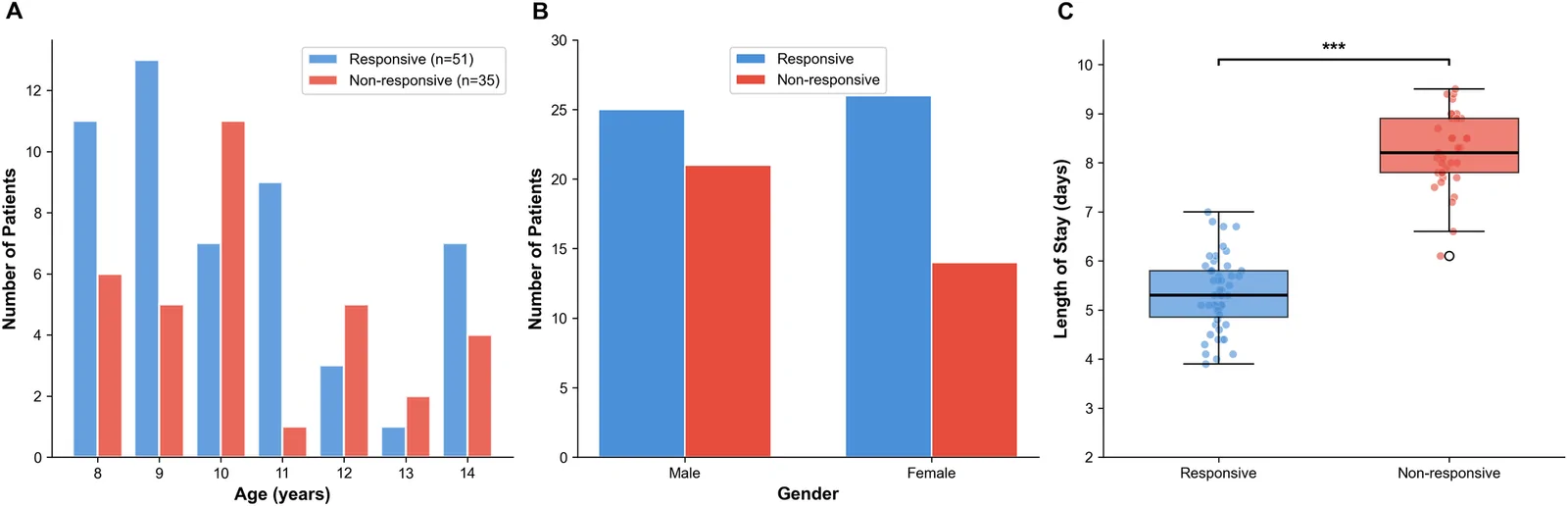
Baseline characteristics of the responsive and non-responsive groups. **(A)** Age distribution. **(B)** Gender distribution. **(C)** Length of hospital stay.

### 3.2 Comparison of laboratory indicators

As shown in Figure 2, the non-responsive group had significantly higher levels of D-dimer (median 0.36 vs. 0.18 mg/L, *P* < 0.001), CRP (*P* = 0.015), LDH (*P* = 0.008), *α*-HBDH (*P* = 0.023), ALT (*P* = 0.030), and ferritin (*P* = 0.002), along with significantly lower albumin levels (*P* < 0.001). Detailed numerical data are provided in Supplementary Table S1. Violin plots for the two primary biomarkers are shown in Figure 3.

**Figure 2:**
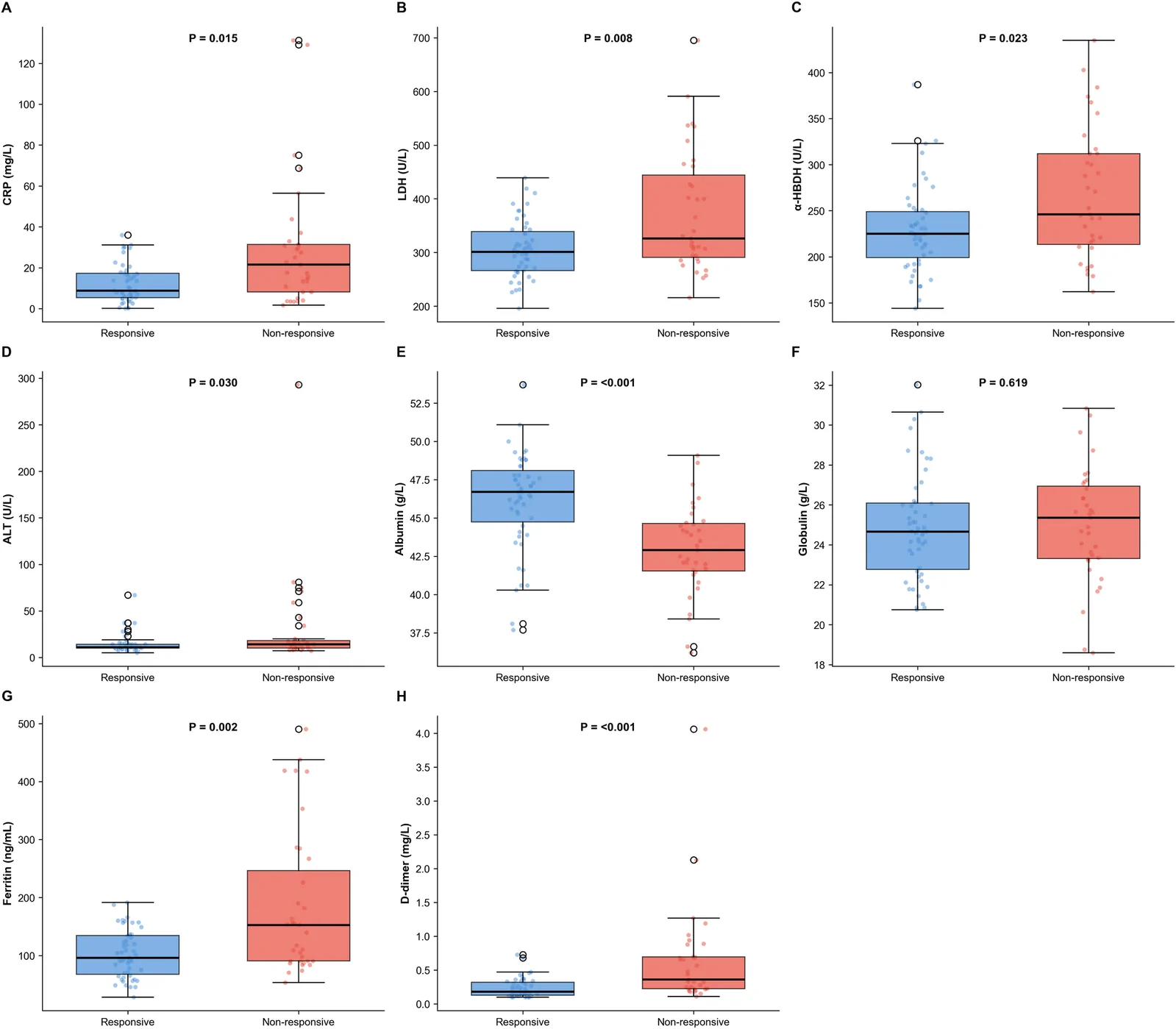
Box plots comparing key laboratory indicators between the responsive and non-responsive groups. **(A)** CRP, **(B)** LDH, **(C)** *α*-HBDH, **(D)** ALT, **(E)** Albumin, **(F)** Globulin, **(G)** Ferritin, **(H)** D-dimer. Individual data points are shown as jittered scatter plots. Significance levels: *P* < 0.05, *P* < 0.01, *P* < 0.001 (Mann-Whitney U test). NLR, neutrophil-to-lymphocyte ratio; CRP, C-reactive protein; LDH, lactate dehydrogenase; *α*-HBDH, *α*-hydroxybutyrate dehydrogenase.

**Figure 3:**
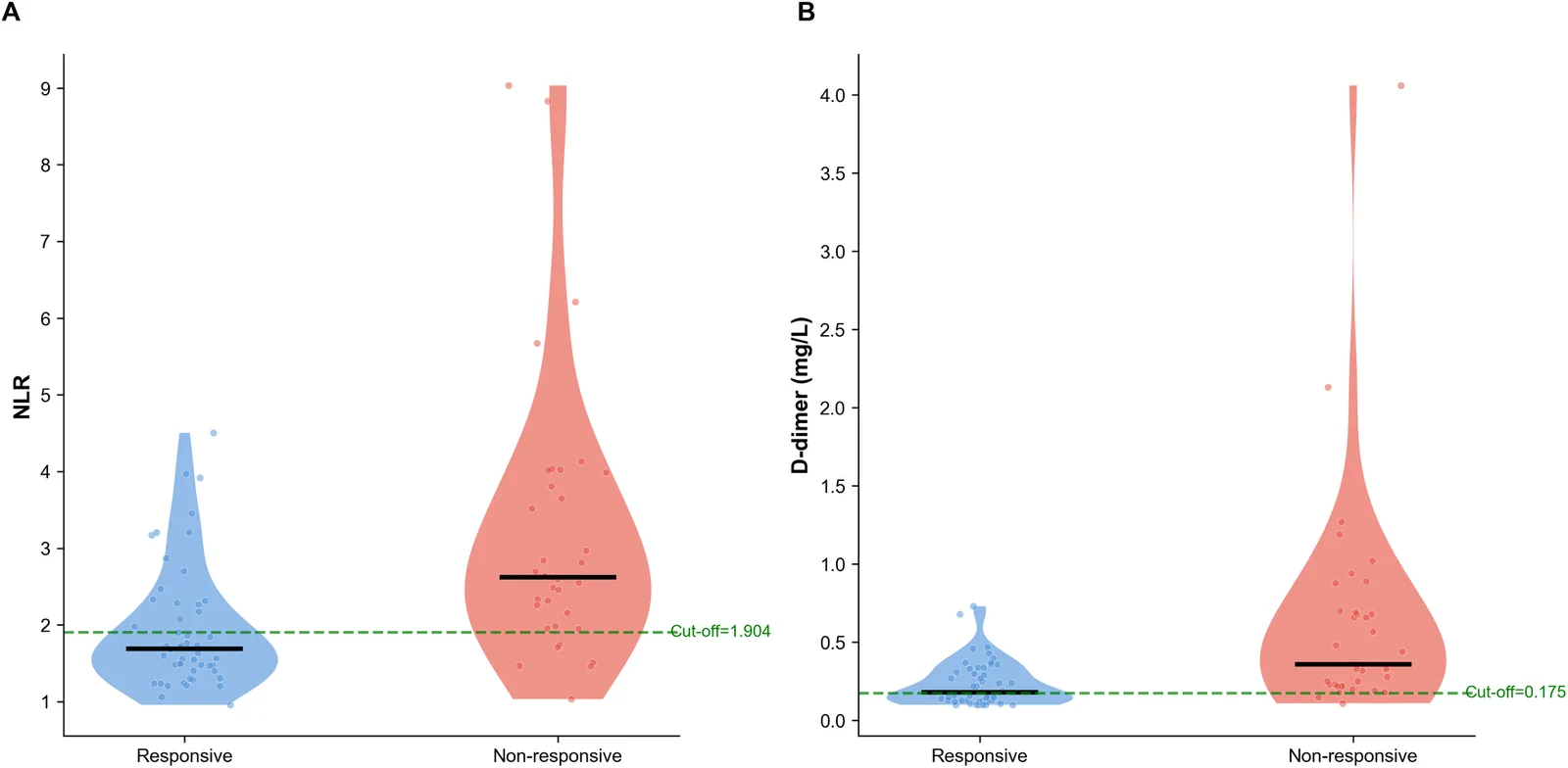
Violin plots showing the distribution of NLR and D-dimer in the responsive and non-responsive groups. **(A)** NLR distribution with optimal cut-off value of 1.904. **(B)** D-dimer distribution with optimal cut-off value of 0.175 mg/L. The horizontal dashed green line indicates the optimal cut-off value. Individual data points are overlaid as scatter plots. The horizontal bar within each violin represents the median.

### 3.3 Multivariate analysis

As illustrated in Figure 4 and Supplementary Table S2, elevated NLR (OR = 2.113, 95% CI: 1.184–3.770, *P* = 0.011) and elevated D-dimer (OR = 148.867, 95% CI: 4.994–4438.007, *P* = 0.004) were identified as significant independent risk factors for treatment failure. Higher albumin levels were protective (OR = 0.762, *P* = 0.009).

**Figure 4:**
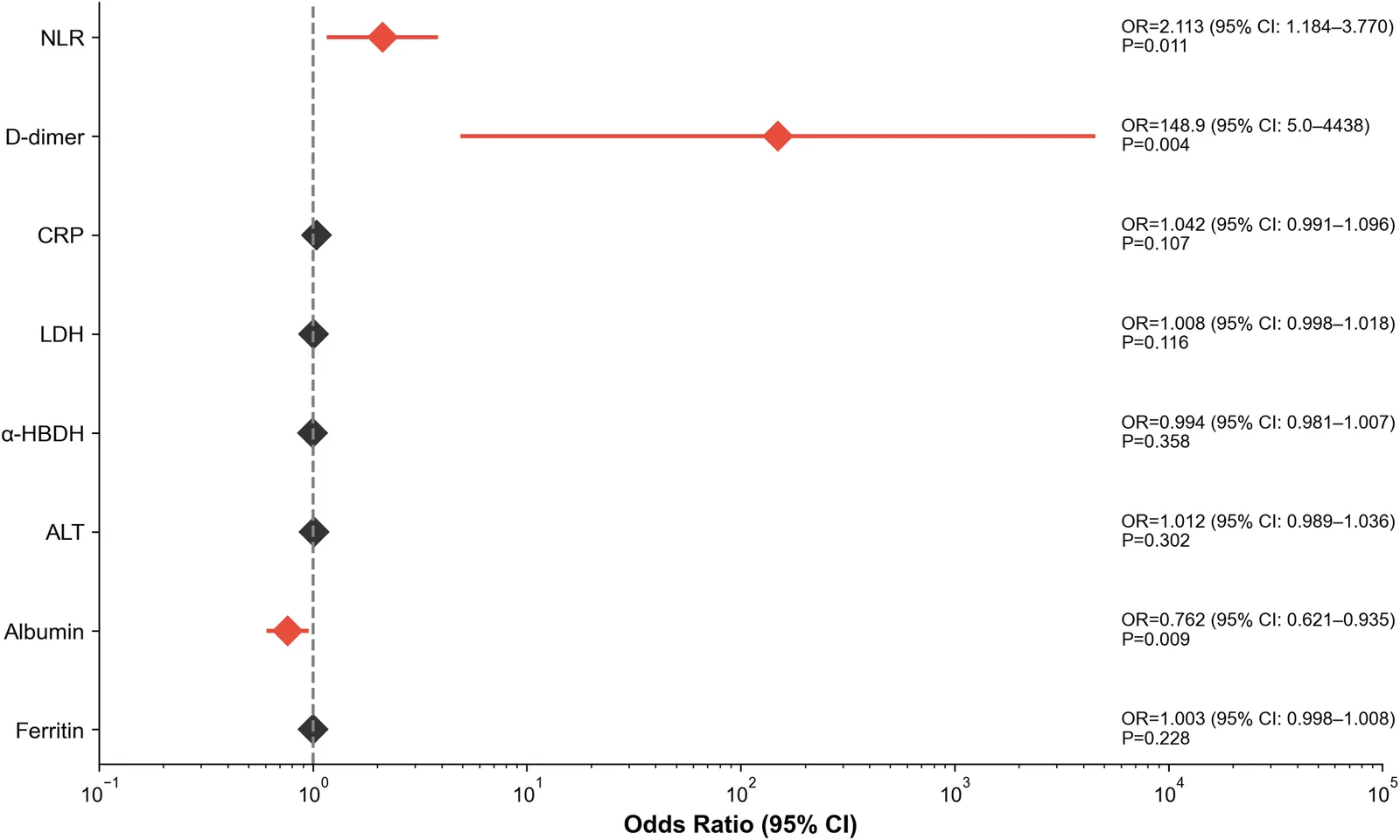
Forest plot illustrating the results of multivariate logistic regression analysis.variables with *P* < 0.05 are shown in red. Diamonds represent odds ratios (OR) and horizontal lines represent 95% confidence intervals (CI). The vertical dashed line indicates OR = 1.

### 3.4 Predictive performance

ROC curve analysis (Figure 5) demonstrated that the AUC for the combined detection of NLR and D-dimer was 0.858 (95% CI: 0.756–0.926), superior to NLR (AUC=0.773) or D-dimer (AUC=0.776) alone. Optimal cut-off values were 1.904 for NLR and 0.175 mg/L for D-dimer.

**Figure 5:**
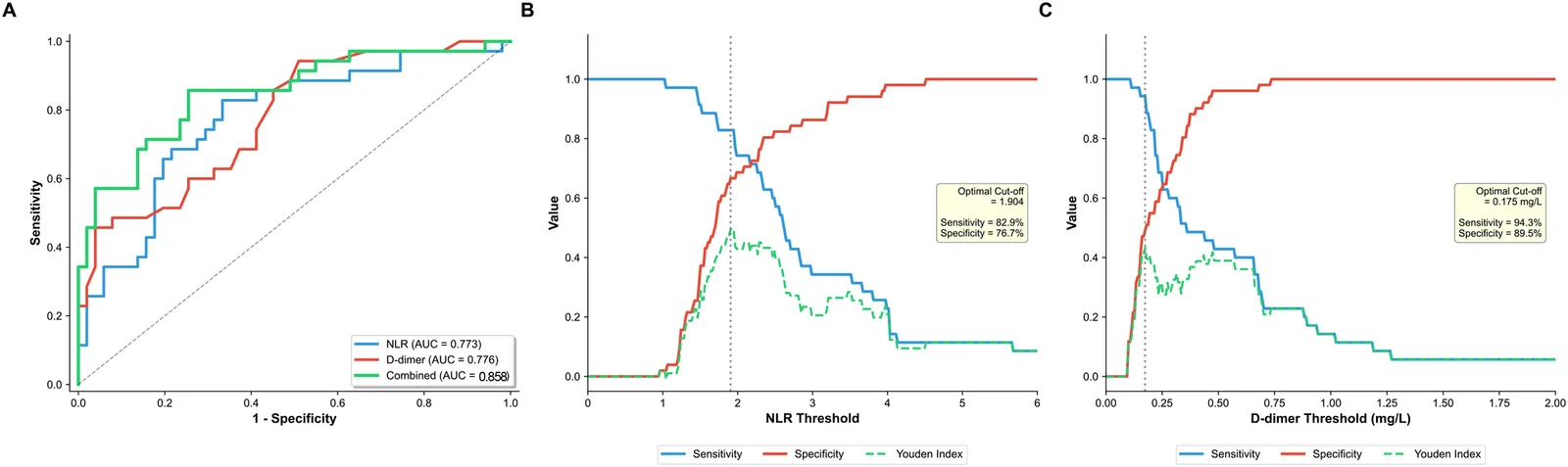
ROC analysis and optimal cut-off determination for NLR and D-dimer. **(A)** ROC curves for NLR, D-dimer, and combined detection. **(B)** Sensitivity, specificity, and Youden index as a function of NLR threshold. **(C)** Sensitivity, specificity, and Youden index as a function of D-dimer threshold. AUC, area under the curve.

### 3.5 Correlation analysis

Spearman correlation analysis (Figures 6, 7) revealed significant intercorrelations among the markers. Both NLR and D-dimer were positively correlated with length of hospital stay (NLR: r = 0.383, *P* = 0.0003; D-dimer: r = 0.477, *P* < 0.0001).

**Figure 6:**
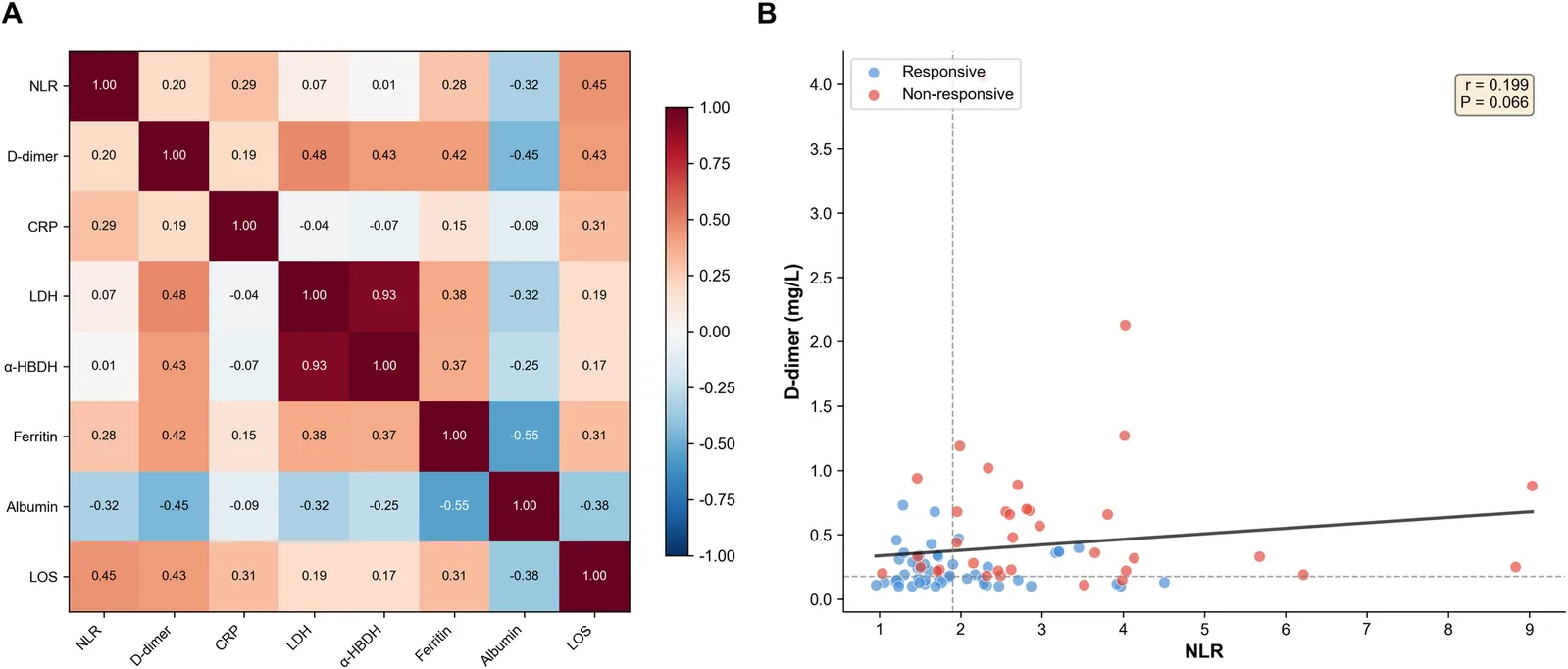
Correlation matrix and relationship between NLR and D-dimer. **(A)** Spearman correlation matrix of key laboratory indicators. Color intensity represents the magnitude of correlation. **(B)** Scatter plot of NLR vs. D-dimer stratified by treatment response. Spearman correlation coefficients and *P* values are annotated.

**Figure 7:**
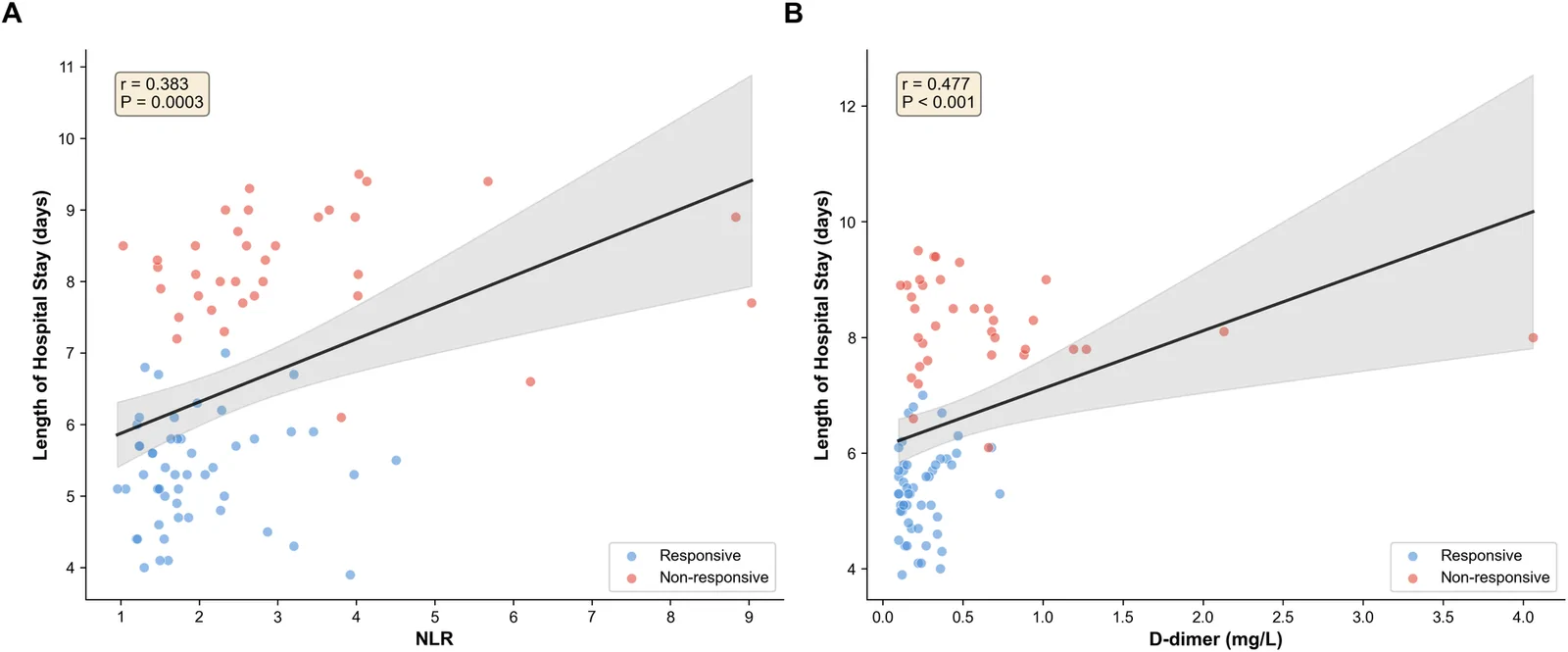
Correlation between biomarkers and length of hospital stay (LOS). **(A)** Correlation between NLR and LOS. **(B)** Correlation between D-dimer and LOS. Spearman correlation coefficients and *P* values are annotated in each panel.

## 4 Discussion

Pediatric MPP is a complex respiratory infection characterized by an increasing incidence and a rising trend of macrolide resistance. While minocycline has demonstrated efficacy against MRMP, the lack of early predictive markers for treatment response poses a significant clinical challenge (14). Traditional evaluation methods relying on pathogen detection and radiographic changes often fail to capture the dynamic inflammatory status and provide timely feedback on drug efficacy. To our knowledge, this study is the first to demonstrate that baseline NLR and D-dimer levels are independent predictors of early clinical response to minocycline in pediatric MPP, and their combined use offers superior predictive value. It is important to distinguish between disease severity and treatment response, as these represent related but distinct clinical concepts. In our study, the grouping was based on treatment response to minocycline (responsive vs. non-responsive) rather than initial disease severity. Some patients with initially severe presentations may respond well to minocycline, while others with seemingly milder disease may not. This distinction is clinically meaningful because it focuses on predicting treatment efficacy rather than simply categorizing disease severity. Our findings suggest that baseline NLR and D-dimer levels may serve as specific predictors of minocycline treatment response, regardless of initial disease severity classification.

Upon Mycoplasma pneumoniae infection, the host immune system mounts a robust inflammatory response. The pathogenesis of MPP is driven not only by direct pathogen invasion but also by an excessive host immune response, often culminating in a ‘cytokine storm’ (15, 16). Neutrophils, as the primary responders, migrate to the infection site and release inflammatory mediators including interleukin-6 (IL-6) and tumor necrosis factor-α (TNF-α), while lymphocytes, responsible for specific immunity, may undergo apoptosis or suppression due to severe infection (17). Consequently, the NLR, representing the balance between innate and adaptive immunity, becomes elevated. Recent studies have highlighted NLR as a reliable predictor of poor prognosis and necrotizing pneumonia in children with MPP (18, 19). Our results showed that the non-responsive group had a significantly higher NLR (median 2.62) compared to the responsive group (median 1.69), and 82.9% of non-responsive group patients exceeded the optimal cut-off of 1.904. Minocycline exerts its antibacterial effect by inhibiting protein synthesis, but it also possesses potent immunomodulatory and anti-inflammatory properties, such as suppressing IL-6 expression and reducing neutrophil chemotaxis (20, 21). In patients who respond well, minocycline effectively dampens the inflammatory cascade, leading to a rapid normalization of the NLR. Conversely, a persistently high baseline NLR suggests an overwhelming hyperinflammatory state that minocycline monotherapy may struggle to control within 48 h, thereby necessitating the early introduction of corticosteroids (13).

In addition to systemic inflammation, non-responsive MPP is frequently complicated by coagulation abnormalities. The pathogen can directly damage vascular endothelial cells and trigger the release of procoagulant factors, leading to a hypercoagulable state and microthrombosis (22). D-dimer, a specific marker of fibrin degradation, accurately reflects this pathological process of thrombosis and secondary fibrinolysis (23). Our study found that D-dimer levels were markedly elevated in the non-responsive group and exhibited strong positive correlations with other severity markers such as LDH and ferritin, suggesting a convergent pathophysiological mechanism involving tissue hypoxia, cellular damage, and coagulation activation. A baseline D-dimer level exceeding 0.175 mg/L demonstrated a high sensitivity of 94.3% in predicting poor response to minocycline. This aligns with recent findings that elevated D-dimer is an independent risk factor for severe MPP and pulmonary embolism (24, 25). Patients with significant coagulation activation are at a higher risk of treatment failure, likely because microvascular thrombosis impedes drug delivery to infected lung tissues and exacerbates local hypoxia and tissue damage, rendering standard antimicrobial therapy insufficient. Crucially, our ROC analysis revealed that the combined detection of NLR and D-dimer achieved an impressive AUC of 0.858, outperforming either marker alone. This synergistic effect is biologically plausible: NLR primarily captures the systemic immune-inflammatory imbalance, whereas D-dimer reflects the extent of endothelial injury and coagulation activation. Together, they provide a comprehensive assessment of the host's pathophysiological state. In clinical practice, monitoring these two accessible biomarkers upon admission can help pediatricians stratify patients. For those with both elevated NLR (>1.904) and D-dimer (>0.175 mg/L), clinicians should maintain a high index of suspicion for minocycline monotherapy failure and consider close monitoring with early initiation of adjunctive corticosteroids if clinical response is inadequate. Further research is needed to evaluate whether anticoagulation strategies may benefit patients with significantly elevated D-dimer levels. This approach is further supported by the significant positive correlations between these markers and the length of hospital stay (NLR: r = 0.383; D-dimer: r = 0.477), demonstrating that higher baseline values are associated with prolonged hospitalization.

The protective role of albumin identified in our multivariate analysis is also noteworthy. Albumin is a negative acute-phase reactant and a marker of nutritional status and hepatic synthetic function. Lower albumin levels in the non-responsive group (median 42.90 vs. 46.70 g/L) may reflect a more severe systemic inflammatory response that consumes albumin, or a compromised physiological reserve that renders patients less capable of mounting an effective response to antimicrobial therapy alone. Hypoalbuminemia has been increasingly recognized as a biomarker of poor prognosis and mortality in critically ill pediatric patients (26). This finding suggests that nutritional support and albumin monitoring may also be relevant in the comprehensive management of pediatric MPP patients who respond poorly to initial therapy. To address the potential impact of extreme D-dimer values on predictive performance, we performed a sensitivity analysis by excluding 2 outliers from the non-responsive group (D-dimer >1.400 mg/L, identified using the IQR method). After exclusion, the AUC for D-dimer alone decreased marginally from 0.776 to 0.763, and the combined AUC decreased from 0.858 to 0.849. These minimal changes confirm the robustness of our predictive model and suggest that the discriminatory power of NLR and D-dimer is not driven by extreme values. Several outlier data points are visible in our figures, particularly in the violin plots of NLR and D-dimer (Figure 3), the scatter plot of NLR vs. D-dimer (Figure 6B), and the correlation plots with length of hospital stay (Figures 7A,B). These outliers represent clinically meaningful cases rather than data errors. Specifically, the patients with extremely high D-dimer levels (>1.0 mg/L) in the non-responsive group were among those who developed the most severe clinical courses, with prolonged fever (>7 days) and complications such as pleural effusion or atelectasis requiring extended hospitalization. Similarly, the outliers with high NLR values (>5.0) corresponded to patients with marked neutrophilia (>8.0 × 10⁹/L) and lymphopenia (<1.0 × 10⁹/L), reflecting an exaggerated inflammatory response. This study has several limitations. First, it is a single-center retrospective study with a relatively small sample size (*n* = 86), which may introduce selection bias. However, our *post-hoc* power analysis demonstrated that this sample size provided robust statistical power (>95%) to detect the observed large effect sizes for both NLR and D-dimer (Supplementary Figure S1), mitigating concerns regarding type II errors. Nonetheless, the findings still need to be validated in larger, multicenter prospective cohorts to ensure broader generalizability. Second, the dynamic changes of NLR and D-dimer (Supplementary Figure S1) during the treatment course were not continuously monitored, limiting our understanding of their kinetic predictive value. Third, the cut-off values derived from this study require validation in independent cohorts. Fourth, the precise timing from illness onset to minocycline initiation was not available in our retrospective dataset. While this information would have provided additional clinical context, the relatively uniform prior macrolide exposure across our cohort (3–5 days) suggests a consistent treatment pathway. Future multi-center, prospective cohort studies with larger sample sizes and longitudinal data collection are warranted to validate our findings and establish standardized clinical pathways for the use of NLR and D-dimer in guiding minocycline therapy for pediatric MPP.

## 5 Conclusion

In conclusion, NLR and D-dimer are valuable, cost-effective, and easily accessible biomarkers for predicting early clinical response to minocycline in pediatric Mycoplasma pneumoniae pneumonia. Elevated baseline levels of NLR and D-dimer are independent risk factors for poor treatment response to minocycline monotherapy. The coefficient of variation (COV) for NLR was lower (41.6%–57.5%) compared to D-dimer (60.9%–115.0%), suggesting that NLR may provide more consistent measurements across patients, although both biomarkers demonstrated significant discriminatory power. The combined detection of these two markers significantly enhances predictive accuracy, with an AUC of 0.858, providing pediatricians with a robust tool for early risk stratification. Implementing this predictive model in clinical practice can facilitate timely adjustments of therapeutic regimens, optimize the use of adjunctive corticosteroids, and ultimately improve the prognosis of children with MPP who respond poorly to initial minocycline therapy.

## Data Availability

The datasets presented in this article are not readily available because retrospective study. Requests to access the datasets should be directed to 13002249716@163.com.

## Supplementary material

Supplementary Figure S1 Presents the statistical power curves for NLR and D-dimer as a function of total sample size. The analysis confirms that the current study sample of 86 patients provides power well above the conventional 80% threshold for both primary biomarkers. Statistical power curves for NLR (left) and D-dimer (right) as a function of total sample size (α=0.05, two-sided). The green dotted horizontal line indicates the 80% power threshold. The filled circle marks the current study sample size (n=86) and its corresponding power.

Supplementary Table S3 Comparison of routine blood parameters between the responsive and non-responsive groups.
