# Supplementary material for: Clinical value of neutrophil-to-lymphocyte ratio and D-dimer in predicting the efficacy of minocycline for pediatric Mycoplasma pneumoniae pneumonia
Source: Front Pediatr. 2026 Sep 17;14:1865687. doi: 10.3389/fped.2026.1865687 (PMC13627409; doi:10.3389/fped.2026.1865687)
Supplement: Supplementary file 1 [file Supplementaryfile1.pdf]

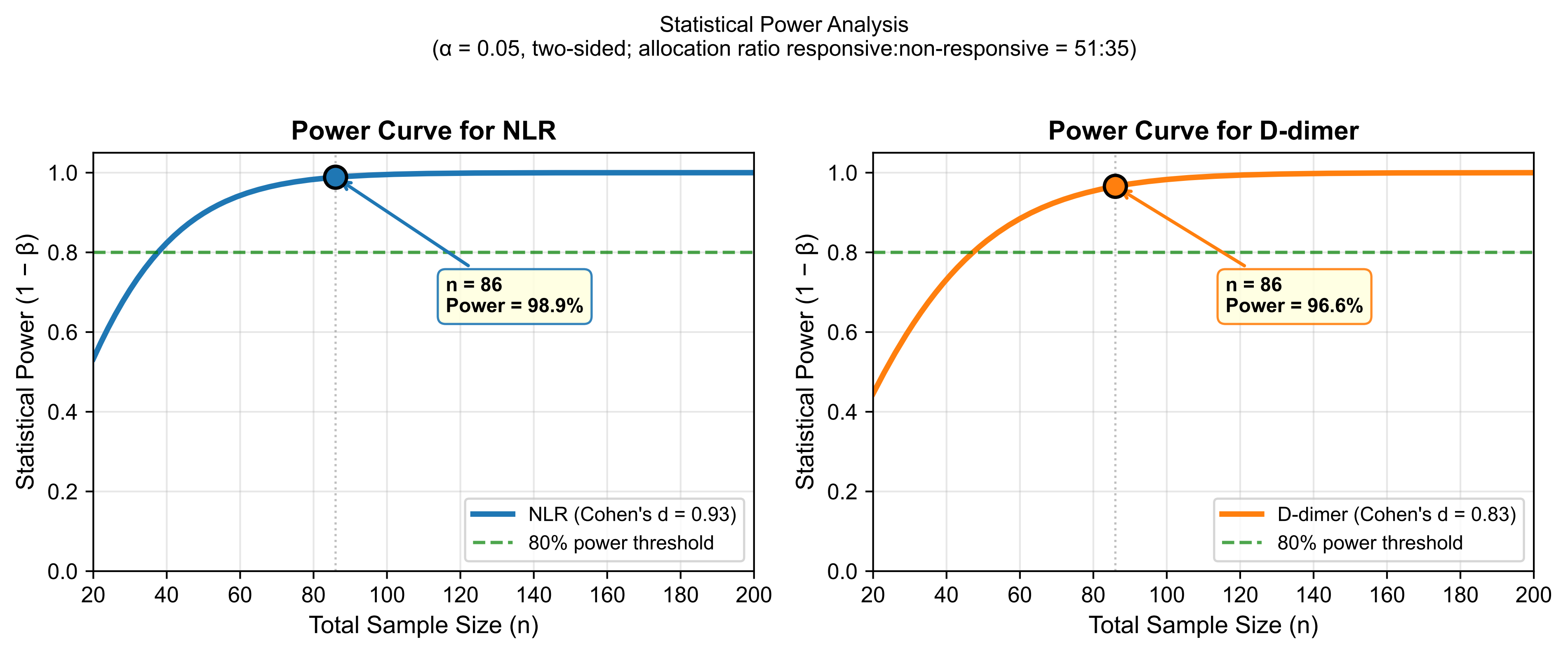

Statistical Power Analysis
(α = 0.05, two-sided; allocation ratio responsive:non-responsive = 51:35)
Power Curve for NLR
Statistical Power (1 − β)
Total Sample Size (n)
n = 86
Power = 98.9%
NLR (Cohen's d = 0.93)
80% power threshold
Power Curve for D-dimer
Statistical Power (1 − β)
Total Sample Size (n)
n = 86
Power = 96.6%
D-dimer (Cohen's d = 0.83)
80% power threshold
