# Supplementary material for: Clinical value of neutrophil-to-lymphocyte ratio and D-dimer in predicting the efficacy of minocycline for pediatric Mycoplasma pneumoniae pneumonia
Source: Front Pediatr. 2026 Sep 17;14:1865687. doi: 10.3389/fped.2026.1865687 (PMC13627409; doi:10.3389/fped.2026.1865687)
Supplement: Supplementary file 2 [file Supplementaryfile2.docx]

**Table S1. Comparison of Blood Cell Analysis Between the Two Groups**

| **Parameter** | **Responsive (n=51)** | **Non-responsive (n=35)** | **P value** |
| --- | --- | --- | --- |
| WBC (×10⁹/L) | 7.16 (6.18, 8.46) | 8.08 (6.09, 9.46) | 0.248 |
| Neutrophil (×10⁹/L) | 4.08 (3.39, 4.96) | 5.02 (3.66, 7.06) | 0.014 |
| Lymphocyte (×10⁹/L) | 2.22 (1.83, 2.70) | 1.76 (1.33, 2.23) | 0.005 |
| Platelet (×10⁹/L) | 222.00 (177.50, 309.00) | 206.00 (162.50, 294.50) | 0.426 |
| NLR | 1.69 (1.44, 2.28) | 2.62 (2.07, 3.90) | <0.001 |
| *Data are presented as median (Q1, Q3). NLR, neutrophil-to-lymphocyte ratio; WBC, white blood cell.* | | | |

**Table S2. Comparison of Inflammatory and Biochemical Markers Between the Two Groups**

| **Parameter** | **Responsive (n=51)** | **Non-responsive (n=35)** | **P value** |
| --- | --- | --- | --- |
| CRP (mg/L) | 8.75 (5.39, 17.30) | 21.50 (8.21, 31.46) | 0.005 |
| LDH (U/L) | 301.00 (266.50, 339.00) | 326.00 (291.00, 444.00) | 0.008 |
| α-HBDH (U/L) | 225.00 (199.00, 249.00) | 246.00 (213.50, 312.00) | 0.023 |
| ALT (U/L) | 11.00 (10.00, 14.00) | 14.00 (10.00, 18.00) | 0.030 |
| Albumin (g/L) | 46.70 (44.75, 48.10) | 42.90 (41.55, 44.65) | <0.001 |
| Globulin (g/L) | 24.66 (22.76, 26.09) | 25.36 (23.32, 26.95) | 0.619 |
| Ferritin (ng/mL) | 95.80 (67.75, 134.45) | 152.50 (90.75, 246.35) | 0.002 |
| D-dimer (mg/L) | 0.18 (0.13, 0.32) | 0.36 (0.23, 0.69) | <0.001 |
| *Data are presented as median (Q1, Q3). CRP, C-reactive protein; LDH, lactate dehydrogenase; α-HBDH, α-hydroxybutyrate dehydrogenase; ALT, alanine aminotransferase.* | | | |

**Table S3. Multivariate Logistic Regression Analysis for Predictors of Treatment Outcome**

| **Variable** | **β** | **Wald** | **OR (95% CI)** | **P value** |
| --- | --- | --- | --- | --- |
| NLR | 0.748 | 6.416 | 2.113 (1.184–3.770) | 0.011 |
| D-dimer | 5.003 | 8.343 | 148.867 (4.994–4438.007) | 0.004 |
| CRP | 0.041 | 2.607 | 1.042 (0.991–1.096) | 0.107 |
| LDH | 0.008 | 2.480 | 1.008 (0.998–1.018) | 0.116 |
| α-HBDH | -0.006 | 0.843 | 0.994 (0.981–1.007) | 0.358 |
| ALT | 0.012 | 1.063 | 1.012 (0.989–1.036) | 0.302 |
| Albumin | -0.272 | 6.836 | 0.762 (0.621–0.935) | 0.009 |
| Ferritin | 0.003 | 1.453 | 1.003 (0.998–1.008) | 0.228 |
| *OR, odds ratio; CI, confidence interval.* | | | | |
